# No substantial change in the balance between model-free and model-based control via training on the two-step task

**DOI:** 10.1371/journal.pcbi.1007443

**Published:** 2019-11-14

**Authors:** Elmar D. Grosskurth, Dominik R. Bach, Marcos Economides, Quentin J. M. Huys, Lisa Holper

**Affiliations:** 1 Department of Psychiatry, University Hospital of Psychiatry, University of Bern, Bern, Switzerland; 2 Department of Psychiatry, Psychotherapy and Psychosomatics, Hospital of Psychiatry, University of Zurich, Zurich, Switzerland; 3 Wellcome Centre for Human Neuroimaging, University College London, London, United Kingdom; 4 Division of Psychiatry and Max Planck UCL Centre for Computational Psychiatry and Ageing Research, University College London, London, United Kingdom; Dartmouth College, UNITED STATES

## Abstract

Human decisions can be habitual or goal-directed, also known as model-free (MF) or model-based (MB) control. Previous work suggests that the balance between the two decision systems is impaired in psychiatric disorders such as compulsion and addiction, via overreliance on MF control. However, little is known whether the balance can be altered through task training. Here, 20 healthy participants performed a well-established two-step task that differentiates MB from MF control, across five training sessions. We used computational modelling and functional near-infrared spectroscopy to assess changes in decision-making and brain hemodynamic over time. Mixed-effects modelling revealed overall no substantial changes in MF and MB behavior across training. Although our behavioral and brain findings show task-induced changes in learning rates, these parameters have no direct relation to either MF or MB control or the balance between the two systems, and thus do not support the assumption of training effects on MF or MB strategies. Our findings indicate that training on the two-step paradigm in its current form does not support a shift in the balance between MF and MB control. We discuss these results with respect to implications for restoring the balance between MF and MB control in psychiatric conditions.

## Author summary

Psychiatric conditions such as compulsion or addiction are associated with an overreliance on habitual, or model-free, decision-making. Goal-directed, or model-based, decision-making may protect against such overreliance. We therefore asked whether model-free control could be reduced, and model-based control strengthened, via task training. We used the well-characterized two-step task that differentiates model-based from model-free actions. Our results suggest that training on the current form of the two-step task does not support a shift in the balance between model-free and model-based strategies. Factors such as devaluation, demotivation or automatization during training may play a role in the missing emergence of a training effect. Future studies could adapt the two-step task so as to separate such factors from decision-making strategies.

## Introduction

Decision-making is suggested to rely on at least two parallel and distinct systems; a retrospectively-driven system based on acquired habits, and a prospective goal-directed system based on deliberate planning [1–7]. Since these two systems sometimes promote different choices, it’s possible to differentiate their relative contribution to decision-making when action-outcome contingencies change; although in reality additional systems may guide decision-making [8] such that increasing reliance on one system does not always decrease reliance on the other [9]. Habits allow performing routines under consistent circumstances with little effort, which can be acquired through reinforcement learning where decisions rewarded in the past are more likely to be repeated in the future [10]. In contrast, goal-directed behavior requires the consideration of potential future outcomes of alternative actions based on the implementation of planned actions and outcomes. In computational terms, these two strategies are described as model-free (MF) and model-based (MB) decision control [1,2,11], respectively. These two strategies are often thought to be employed in parallel but the arbitration between them as determined by situations, actions and outcomes, has to be learned by exploration of the state-transition prediction error [12].

A wealth of evidence suggests that the two systems are implemented in partly dissociable but overlapping cortico-striatal circuits in the brain [13]. Neuroimaging studies using functional magnetic resonance imaging (fMRI) showed contributions of dorsolateral striatum (DLS), dorsomedial striatum (DMS) and prefrontal cortex (PFC) [14–17]. DLS appears to be predominantly involved in the formation of MF decisions [18–20] with connections to premotor cortex (PMC). These areas encode stimulus-response pairs but without representation of decision outcomes [20]. In contrast, DMS encodes MB decisions [21–25] reflected in an extensive level of connections with orbitofrontal, ventromedial (vmPFC) and dorsolateral PFC (dlPFC) [26]. These areas encode the relationship between states, actions and outcomes [20]. Finally, inferior lateral PFC (ilPFC) has been suggested to represent the neural signature of an arbitrator responsible for the balance between the two strategies [12,27].

An imbalance between the two systems in favor of the MF system has been related to maladaptive choices in psychiatric disorders [28,29]. For example, excessive overreliance on habitual control has been shown in obsessive-compulsive disorder (OCD) [24,25] when rigid habits result in inadequate, repetitive and self-deleterious compulsive actions. Behavioral control may then become insensitive towards negative long-term consequences [30], the latter has been shown to correlate with altered prefrontal signals [31]. Beyond OCD, deficits in goal-directed behavior have also been reported in patients with addiction [24,32–35], social anxiety [36,37] and schizophrenia [38–40]. The finding of similar MB deficits across different psychiatric disorders enforces the idea of a trans-diagnostic symptom approach [41].

**Fig 1:**
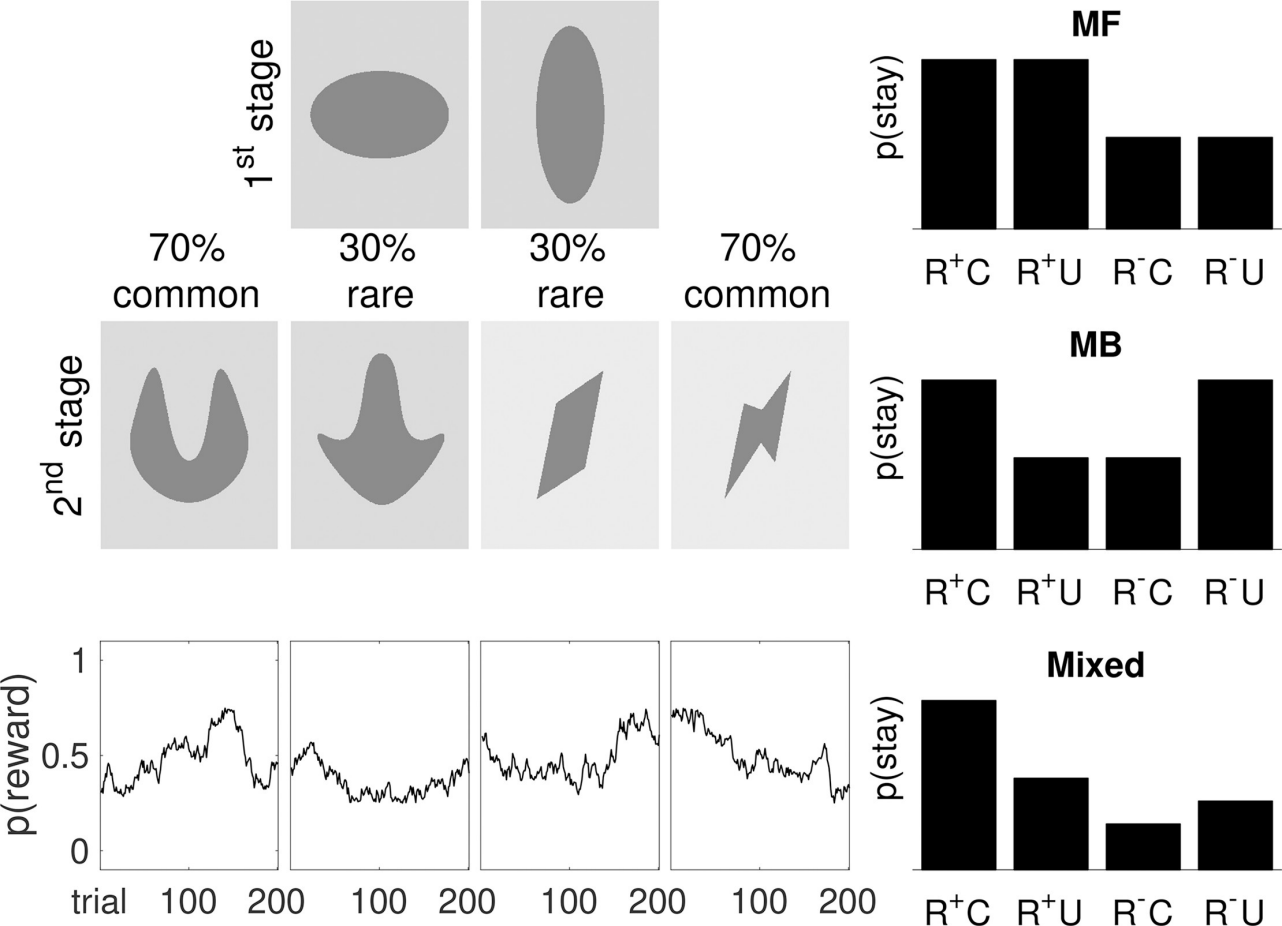
**(Left) Two-step task.** Each 1^st^ stage led to a 2^nd^ stage in 70% of trials (*common transition*) and in 30% of trials to another 2^nd^ stage (*uncommon transition*). Reward probabilities (*p*(reward)) for each 2^nd^ stage fluctuated across trials between 25% and 75% according to Gaussian random walks [1]. **(Right) Model predictions.** Predictions on MF versus MB learning for the probability to repeat the choice from the previous trial (p(stay)) as a function of reward (R^+^ = rewarded vs. R^-^ = unrewarded) and transition (C = common vs. U = uncommon) at the previous trial. MF predicts a main effect of ‘reward’ and no effect of ‘transition’, whereas MB predicts an interaction effect of ‘reward * transition’. Mixed effects of both MB and MF are typically identified in the two-step task [1]. Figure adapted from [67].

Based on the assumption that overreliance on MF control can result in harmful habits and that MB learning is protective against the formation of those habits [42], the question arises of whether MB strategies can be strengthened by training. The well-characterized two-step task [1] (**Fig 1**) that promises to differentiate MB from MF learning through the implementation of parametric decision variables [43], is a likely candidate for such a training approach. The two-step task requires continuously updating action values for optimal behaviour under randomly fluctuating reward probabilities. It may therefore encourage goal-directed learning [1] and does not induce overtraining, which in animals has been shown to encourage MF strategies [44]. Indeed, a previous study by Economides et al. [9] suggested that short-term training on the two-step paradigm (768 trials across three consecutive days) improves MB control while leaving MF control unaffected, however only when participants were placed under additional cognitive load via a secondary task. The present work hypothesized that more intensive training (1005 trials across five sessions, each separated by a week) on the same task [1] may both reduce MF and strengthen MB control in the long-term. In addition, we aimed to evaluate whether behavioral training effects would be accompanied by changes in prefrontal brain activations. In order to facilitate future clinical studies, we utilized functional near infrared spectroscopy (fNIRS), which is more readily available and easier to integrate into clinical settings than, for example, fMRI.

## Material and methods

### Ethics statement

All participants gave written informed consent. The study was approved by the governmental ethics committee (KEK Zurich) and conducted in accordance with the Declaration of Helsinki.

### Participants

Thirty-three healthy participants (age 25.5 ± 4.4 mean ± STD, 17 females) were recruited at the University of Zurich. Exclusion criteria were psychiatric or neurological disorders or current medication.

### Experimental protocol

We used the two-step task by Daw et al. [1] (**Fig 1**) programmed in MATLAB (The MathWorks, MA) [45] with the Psychophysics Toolbox [46]. The task consisted of 201 trials, each comprising two stages. In the 1^st^ stage, participants chose between two options (‘states’) represented by geometrical coloured shapes. In the 2^nd^ stage, participants were presented with either of two more states which were rewarded with money (0.2 Swiss Francs) or not (zero). Which 2^nd^ stage state was presented depended probabilistically on the 1^st^ stage choice according to a fixed common (70% of trials) and uncommon (30% of trials) transition scheme. In order to encourage learning, reward probability for each 2^nd^ stage stimulus fluctuated slowly and independently by adding independent Gaussian noise (mean 0, SD .025), with reflecting boundaries at .25 and .75 [1].

Trials were separated by an inter-trial-interval of random duration between 5–11 seconds. If participants failed to make choices within 2 seconds, the trial was excluded from analysis.

The goal of the task for the participants was to identify the rewarding 2^nd^ stage state and make the 1^st^ stage choice accordingly. To achieve this, participants were required to build an internal model of both 1^st^ stage transitions and of 2^nd^ stage reward probabilities.

Prior to the first training session, participants underwent extensive self-paced computer-based instructions and performed 50 practice trials (approx. 20 minutes). Instructions gave detailed information about the task structure, the fixed transition probabilities between 1^st^ and 2^nd^ stage and the varying reward probabilities at the 2^nd^ stage. Participants were instructed to win as much reward as they could and that they would be paid depending on their cumulative performance across a randomly drawn one-third of all trials in each session. Each participant performed five training sessions on five days (total of 1005 trials) separated by a week.

### fNIRS instrumentation

**Fig 2:**
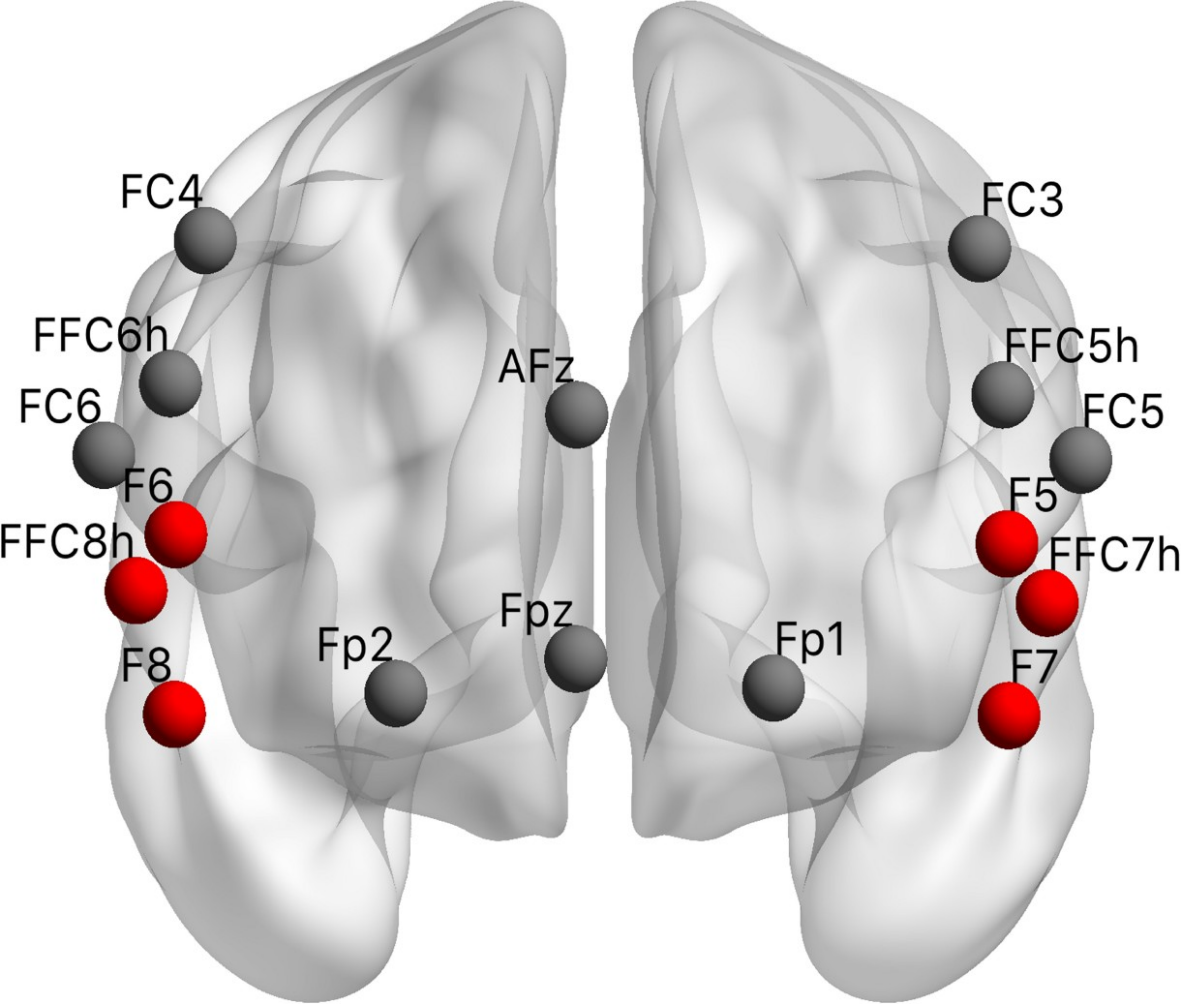
fNIRS setup. Regions of interest corresponding to the MB system in vmPFC (Fpz, Fp1, Fp2, AFz) and dlPFC (FC5, FC6, FFC5h, FFC6h, FC4, FC3) and the arbitrator in ilPFC (red, F7, F8, FFC7h, FFC8h, F5, F6) following previous work [27] (**S1 Table**).

A NIRSport instrument (LLC NIRx Medical Technologies) was used to record cortical hemodynamic responses during task performance in each session. Regions of interest were selected to correspond to the vmPFC (Fpz, Fp1, Fp2, AFz) and dlPFC (FC5, FC6, FFC5h, FFC6h, FC4, FC3) which have both been suggested to represent pure MB strategies [27], and the ilPFC (F7, F8, FFC7h, FFC8h, F5, F6) that is thought to encode the arbitrator between the MF and the MB system [27] (**Fig 2, S1 Table**). Regions corresponding to the MF system, such as DLS [27], were not recorded because fNIRS has a limited depth of tissue penetration and can therefore not record subcortical areas.

The fNIRSport system utilizes time-multiplexed dual-wavelength light-emitting diodes (wavelengths 760 nm and 850 nm) with photo-electrical detectors (Siemens, Germany). Sources and detectors were placed in a head cap providing a source-detector distance of approximately 30 mm. Custom made short channels (approx. 10 mm) were used to remove superficial tissue contributions. Functional recordings acquisitioned using LabVIEW (National Instruments, Austin, TX, USA) were pre-processed including baseline correction, detrending and band-pass filtering [47]. Data were visually inspected for motion artifacts (“steps” and “spikes”) that were removed in 15 participants using NIRSlab [48]. Concentration changes of oxy- (Δ[O_2_Hb]) and deoxy- (Δ[HHb]) hemoglobin were calculated by use of the Beer-Lambert Law (absorption coefficients (μ_a_) for O_2_Hb: μ_a_(760 nm) = 1486, μ_a_(850 nm) = 2526, for HHb: μ_a_(760 nm) = 3843, μ_a_(850 nm) = 1798; differential pathlength factor (DPF): DPF(760 nm) = 7.25, DPF(850nm) = 6.38). Total hemoglobin Δ[tHb], computed as the sum of Δ[O_2_Hb] and Δ[HHb], was chosen as primary parameter of interest because it is thought to be more specific for mapping cerebral activity [49,50]. Trial-by-trial estimates of Δ[tHb] were derived using the general linear model (GLM) approach [51,52] by convolving a stick function at actual choice with a hemodynamic response function for NIRS data [53]. We only modelled 1^st^ stage choices because hemodynamic responses to 1^st^ and 2^nd^ stage choices could not be unambiguously separated due to the short inter-trial-interval [52,54].

### Data analysis

Data analysis was performed to assess overall training outcomes (response times and reward rates) followed by analyses based on logistic and linear mixed-effects (LME) (behavioral choice and hemodynamic responses) and computational modelling (behavioral choice) as well as a simulation to relate LME and modelling.

### Response times and reward rates

Training effects on response times and reward rates were assessed using repeated measures ANOVA with Bonferroni correction. In case of significant main effects, polynomial contrasts were assessed.

### LME regression

We first analyzed stay-versus-switch behavior on 1^st^ stage choices of each trial to dissociate the relative influence of MF and MB control. As mentioned above, MF learning predicts that rewarded choices will lead to a repetition of that choice irrespective of a following common or uncommon transition, because the transition structure is not considered (**Fig 1**); a reward after a uncommon transition would therefore adversely increase the value of the chosen 1^st^ stage state without updating the value of the unchosen state. By contrast, MB strategy predicts an interaction between transition and reward, because an uncommon transition inverts the effect of a subsequent reward (**Fig 1**); a reward after an uncommon transition would therefore increase the probability to choose the previously unchosen 1^st^ stage state. Hence, MF behavior has been suggest to be quantifiable as main effect of ‘reward’ and no effect of ‘transition’, whereas MB behavior may be quantified as interaction effect of ‘reward * transition’ [55].

LME regression was fitted using the glmer function from the lme4 package [56] in R [57] for the effects of ‘reward’ (coded as *rewarded* 1, *unrewarded* -1), ‘transition’ (coded as *common* 1, uncommon -1) and their interaction ‘reward * transition’ (choice ~ reward * transition + (1 + reward * transition | subject)) in predicting each trial’s choice (coded as *switch* 0 and *stay* 1, relative to the previous trial) with states being treated independently [58]. Following previous work [43], we also included an additional random ‘correct’ predictor capturing the tendency of the agent to repeat correct choices, in order to prevent differences in action values at the start of the trial from appearing as a spurious loading on the transition-outcome interaction predictor [43]; the inclusion of this predictor only marginally affected results. The function anova from the lme4 package was used to extract F-stats and p-values. To graphically demonstrate training effects on the balance between MF and MB control, the LME coefficients indexing MF (effect of ‘reward’) and MB (interaction of ‘reward * transition’) control were illustrated following previous work [9,55].

Analogous to the behavioral choice data, the scaled hemodynamic responses in vmPFC, dlPFC and ilPFC were fitted using linear mixed-effects (LME) regression based on the lmer function from the lme4 package [56] in R [57]. The relation between the behavioral and brain LME coefficients was assessed using Pearson product moment correlation.

### Computational model

Since LME one-step effects reflect not only expression of MF and MB strategies but also parametric changes within the two systems and may therefore mislead interpretations [59], we compared the LME results with computational modelling of the two-step task [1,60].

Based on the original hybrid model by Daw et al. [1], we compared eight different model variants as implemented in the Emfit toolbox (https://www.quentinhuys.com/pub/emfit/) in MATLAB (MathWorks, MA) [61] using priori Bayesian model comparison. The model with the best fit to the data was a variant of the original model which has two separate betas, one for the MB system and one for the MF system, rather than a weight explicitly trading off the two components as the weighting parameter (ω) in Daw et al. [1] (**S2 Table**). Model selection was based on the lowest integrated Bayesian information criterion (iBIC) score which is the sum of integrals over the individual parameters [60].

For details on the models see Huys et al. [60]. In brief, the MF strategy is computed using the SARSA (λ) temporal difference (TD) model, which learns the task by strengthening or weakening associations between 1^st^ stage states and 1^st^ stage actions depending on whether the action is followed by a reward or not [62]. It simply predicts that 1^st^ stage actions that resulted in a reward are more likely to be repeated in the next trial with the same initial state [1]. This is quantified by calculating the value for each state-action pair at each stage of each trial with the model allowing different learning rates α_1_ and α_2_ for 1^st^ and 2^nd^ stages, respectively. The reinforcement eligibility parameter (λ) determines the update of 1^st^ stage actions by the 2^nd^ stage prediction error (Q_TD_), with λ = 1 being the case of **Fig 1 (MF)** in which only the final reward is important, and λ = 0 being the purest case of the TD algorithm in which only the 2^nd^ stage value plays a role. On the other hand, MB strategy uses an internal model of the task structure to determine 1^st^ stage choices that will most likely result in a reward [1]. It thus considers which 2^nd^ stages are most frequently rewarded in recent trials and selects 1^st^ stage actions that most likely led there. This is quantified by mapping state-action pairs to the transition function, the common or the uncommon transition. The action value (Q_MB_) is thus computed at each trial from the estimates of the rewards and transition probabilities (**Fig 1, MB)**. Choice randomness is reflected in the softmax inverse temperature parameter at the 2^nd^ stage (β_2_) that controls how deterministic choices are and *p* captures perseveration (*p* > 0) or switching (*p* < 0) in 1^st^ stage choices. Finally, contrary to the original model [1] that uses a weighted sum (Q_NET_) of MF and MB strategies (weighting parameter, ω) at the 1^st^ stage, the model variant has two separate betas, one for the MB system and one for the MF system. The model variant thus tests whether the assumption of the original model [1] that the two approaches coincide at the 2^nd^ stage (i.e., that Q_MB_ = Q_TD_, Q_NET_ = Q_MB_ = Q_TD_ at the 2^nd^ stage) holds true.

Taken together, the hybrid model variant outputs seven free parameters: bMB and bMF, the betas governing the tradeoff between MB and MF actions; the inverse temperature parameter at the 2^nd^ stage (β_2_); the 1^st^ (α_1_) and 2^nd^ (α_2_) stage learning rates; the reinforcement eligibility parameter (λ); and *p*, which captures first-order perseveration. All five training sessions across participants (N = 100) were fitted simultaneously with all data treated as derived from the same prior distribution.

The bounded model parameters were transformed to an unconstrained scale via exponential transformation for parameters bMB, bMF, β_2_ according to Eq 1 and via sigmoid transformation for parameters α_1_, α_2_, and λ according to Eq 2:

x=exp(x) (1)

$$x=\frac{1}{1+\exp\left(-x\right)}$$ (2)

To assess training effects on the seven model parameters one-way repeated measures ANOVA with Bonferroni correction was performed; in accordance with the assumption of the fitting procedure that sessions were drawn from the same Gaussian prior distribution. In case of significant main effects, polynomial contrasts were assessed. To validate the goodness of fit, the subject-specific BIC [63] was compared between sessions using repeated measures ANOVA.

To assess test-retest reliability of the model parameters, the Intraclass Correlation Coefficient (ICC) was used. ICC were computed as type ICC(2,k) according to the Shrout and Fleiss convention [64], i.e., a two-way random-effects model with absolute agreement. P-values of the hypothesis test ICC = 0 based on alpha level p < 0.05 were reported. ICC < 0.4, 0.4–0.75, > 0.75 are considered poor, moderate and excellent reliability, respectively [65].

To assess test-retest repeatability, the Coefficient of Variation (CV), defined as the ratio of the standard deviation to the absolute mean, was calculated [66]. CV is a measure of precision with higher values indicating greater level of dispersion expressed in percentage (%) and therefore allows for comparison between model parameters independent of their units (in contrast to the ICC that is based on units).

### Simulating the relation between LME and modelling

To evaluate the relation between LME and modelling, simulation was conducted to assess how LME regression captures the seven parameters (bMB, bMF, β_2_, α_1_, α_2_, λ, p). For this purpose, data were generated for 1000 subjects with each 201 trials by independently changing each of the seven parameters within the distribution of the untransformed values obtained from the actual data (5^th^, 25^th^, 50^th^, 75^th^, 95^th^ percentile, across sessions S1-S5) while keeping the remaining parameters constant at the median (**S4 Table**). Based on the simulation, we estimated the relative parameter-specific changes in LME coefficients for MF control (‘reward’ effect) and MB control (‘reward * transition’ interaction) for each parameter. This was done by computing the correlation between the independent parameter changes and the induced changes in LME coefficients and describing them as parameter-specific correlation indices (MF_CI_ and MB_CI_).

## Results

Twenty participants (mean ± STD = 24.9 ± 3.1 age, 9 females) completed five training sessions (mean duration 51.8 minutes, repeated measures ANOVA F_4,76_ = 1.82, p = 0.133). 13 additional participants were excluded because of non-adherence to at least one training session (n = 12) or due to technical problems (n = 1, failure of data synchronization).

### Response times and reward rates

**Fig 3:**
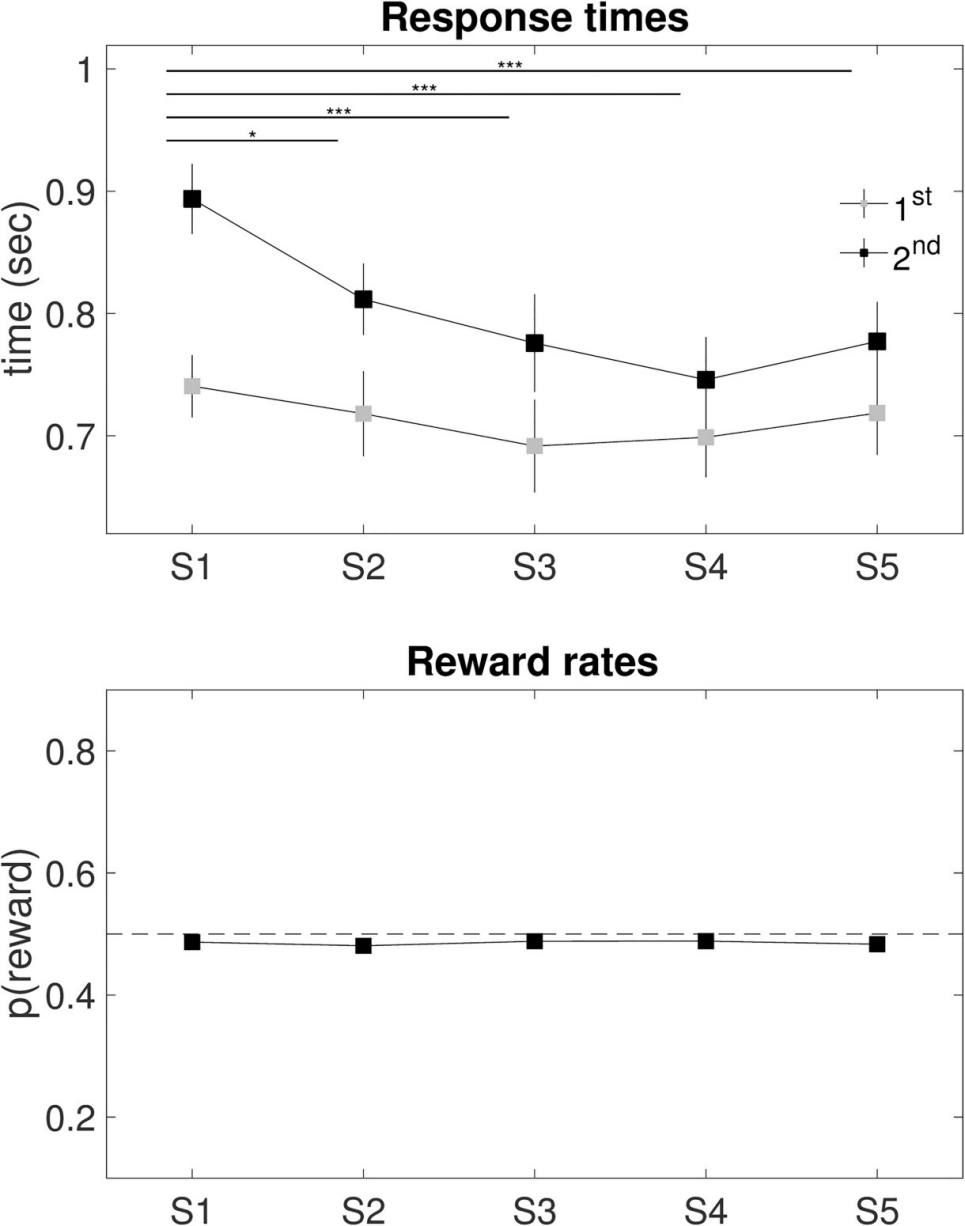
Response times and reward rates. Training effects were observed on 2^nd^ stage (but not 1^st^ stage) response times, which were longer in S1 compared to any of S2-S5. No training effect was observed on overall reward rates. Error bars represent standard error of the mean. Dashed horizontal line indicates chance level. See **Table 1** for statistics.

Response times in the 2^nd^ stage (repeated measures ANOVA F_4,76_ = 10.90, p < 0.001), but not in the 1^st^ stage (F_4,76_ = 2.01, p = 0.101), revealed significant change over time, with S1 RTs longer than in any of S2-S5. Reward rates revealed no training effects (repeated measures ANOVA F_4,76_ = 0.13, p = 0.971), in line with previous work [43,67] (**Table 1, Fig 3**).

**Table 1:** Response times and reward rates. Repeated measures ANOVA assessing training effects on response times (RT) and reward rates. Significant results on an alpha level p < 0.05 are highlighted (bold). See **Fig 3** for illustration.

		1^st^ stage RT	2^nd^ stage RT	Reward
**Main effect**	**F**_**4,76**_	2.01	10.90	0.13
	**p-value**	0.101	**0.000**	0.971
**Post-hoc**	**S1 vs. S2**	1.000	**0.012**	1.000
	**S1 vs. S3**	0.125	**0.000**	1.000
	**S1 vs. S4**	0.320	**0.000**	1.000
	**S1 vs. S5**	1.000	**0.000**	1.000
	**S2 vs. S3**	1.000	1.000	1.000
	**S2 vs. S4**	1.000	0.086	1.000
	**S2 vs. S5**	1.000	1.000	1.000
	**S3 vs. S4**	1.000	1.000	1.000
	**S3 vs. S5**	1.000	1.000	1.000
	**S4 vs. S5**	1.000	1.000	1.000

### LME regression

**Fig 4:**
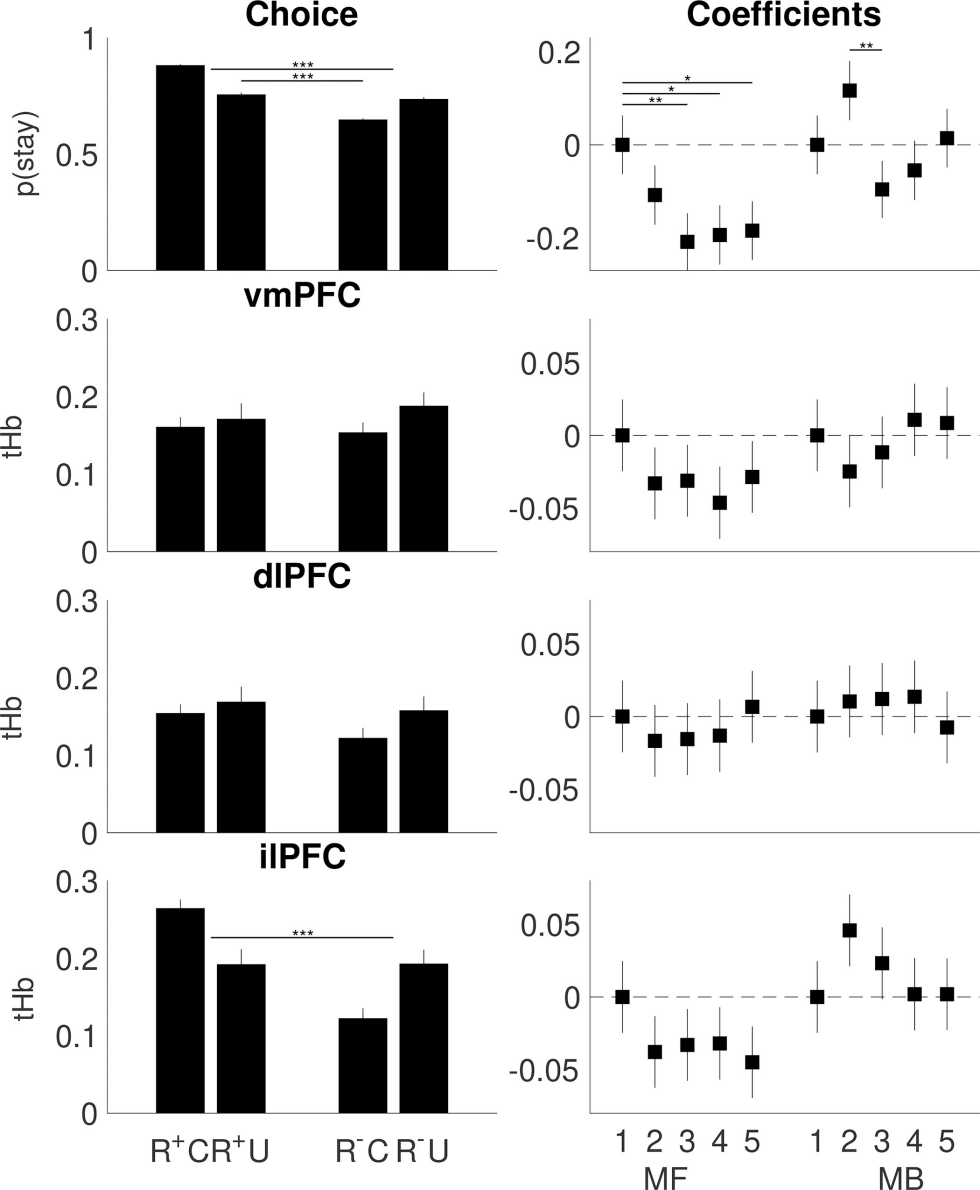
**LME main effects (Left).** Each bar represents the stay probability (p(stay)) or mean tHb response across all participants and all sessions. Error bars represent standard error of the mean. Behavioral choice revealed ‘reward’ effects (R+ = rewarded vs. R- = unrewarded) (solid line with significance asterisks) and ‘reward * transition’ interactions (C = common vs. U = uncommon) (dashed line with significance asterisks), while ilPFC revealed a ‘reward’ effect (solid line with significance asterisks). See **S1 Fig** for details. **LME coefficients (Right).** Between sessions, behavioral choice revealed ‘reward * session’ and ‘reward * transition * session’ interactions, whereas no such effects were found on vmPFC, dlPFC and ilPFC. Error bars represent standard error of the estimate. Significant post-hoc comparisons on the interaction effects are Bonferroni corrected and highlighted (*). See **Table 2** for statistics.

Across sessions, LME regression of behavioral choice showed effects of ‘reward’ (ANOVA F_1,19758_ = 32.50, p < 0.001) and a ‘reward * transition’ interaction (F_1,19758_ = 40.80, p < 0.001) (**Table 2, Fig 4**). Both were affected by training. We observed a ‘reward * session’ interaction (F_4,19758_ = 15.30, p = 0.004), which was mainly due to a decrease from S1 to S5 (post-hoc tests: S1 vs. S3 p = 0.006, S1 vs. S4 p = 0.019, S1 vs. S5 p = 0.028). Furthermore, there was a ‘reward * transition * session’ interaction (F_4,19758_ = 13.26, p = 0.010), mainly due to an increase in S2 only (‘S2 vs S3 p = 0.005). We also found a main effect on ‘transition * session’ (F_4,19758_ = 9.70, p = 0.046), which was however less pronounced than the other effects.

Across sessions, LME regression of ilPFC response showed an effect of ‘reward’ (F_1,19758_ = 12.84, p < 0.001) but no ‘reward * transition’ interaction (F_1,19758_ = 1.18, p = 0.278). Both were not affected by training (‘reward * session’ F_4,19758_ = 3.94, p = 0.414; ‘reward * transition * session’ F_4,19758_ = 5.18 p = 0.269) (**Table 2, Fig 4**). The resulting ilPFC LME regression coefficients correlated significantly with those obtained for behavioral choice (r = 0.83, p = 0.003), supporting a correspondence between behavioral choice and ilPFC which has been thought to encode an arbitrator between the MF and MB system [27]. The two other regions, vmPFC and dlPFC, both thought to reflect MB control [27], revealed no relevant effects and correlated less with behavioral choice (vmPFC r = 0.58, p = 0.081, dlPFC r = 0.43, p = 0.210).

**Table 2:** LME. **Top.** ANOVA (F-stats and p-values) of the logistic and linear mixed-effects regression on behavioral choice, vmPFC, dlPFC and ilPFC. Degrees of freedom (DF). **Bottom.** LME coefficients (COEF with standard error, SE, and p-values) are shown in comparison with the reference session S1. For post-hoc comparisons see **Fig 4**.

			**CHOICE**		**vmPFC**		**dlPFC**		**ilPFC**	
		**DF1, 2**	**F**	**p**	**F**	**p**	**F**	**p**	**F**	**P**
**LME ANOVA**	Intercept	1, 19758	82.41	0.000	15.30	0.000	75.72	0.000	140.01	0.000
	Reward	1, 19758	32.50	**0.000**	2.07	0.150	1.07	0.300	12.84	**0.000**
	Transition	1, 19758	22.14	**0.000**	0.85	0.357	0.56	0.456	0.03	0.868
	Session	4, 19758	30.87	**0.000**	2.87	0.580	6.78	0.148	2.29	0.683
	Reward * Transition	1, 19758	40.80	**0.000**	0.16	0.689	0.00	0.968	1.18	0.278
	Reward * Session	4, 19758	15.30	**0.004**	3.77	0.438	1.43	0.839	3.94	0.414
	Transition * Session	4, 19758	9.70	**0.046**	6.16	0.187	4.66	0.324	0.51	0.972
	Reward * Transition * Session	4, 19758	13.26	**0.010**	2.85	0.583	1.08	0.898	5.18	0.269
			**COEF (SE)**	**p**	**COEF (SE)**	**p**	**COEF (SE)**	**p**	**COEF (SE)**	**P**
**LME COEFFICIENTS**	Intercept		1.46 (0.16)	0.000	0.07 (0.02)	0.000	0.15 (0.02)	0.000	0.21 (0.02)	0.000
	Reward		0.65 (0.11)	0.000	0.03 (0.02)	0.152	0.02 (0.02)	0.301	0.06 (0.02)	0.000
	Transition		0.26 (0.06)	0.000	-0.02 (0.02)	0.358	-0.01 (0.02)	0.457	0 (0.02)	0.868
	Session2		0.01 (0.06)	0.860	0.03 (0.02)	0.277	-0.02 (0.02)	0.456	-0.02 (0.02)	0.364
	Session3		-0.27 (0.06)	0.000	0.03 (0.02)	0.257	-0.03 (0.02)	0.180	-0.02 (0.02)	0.416
	Session4		-0.01 (0.06)	0.930	0.04 (0.02)	0.108	0.03 (0.02)	0.254	-0.04 (0.02)	0.136
	Session5		-0.01 (0.06)	0.844	0.03 (0.02)	0.235	-0.01 (0.02)	0.718	-0.02 (0.02)	0.502
	Reward * Transition		0.41 (0.06)	0.000	0.01 (0.02)	0.689	0 (0.02)	0.968	0.02 (0.02)	0.278
	Reward * Session2		-0.11 (0.06)	0.090	-0.03 (0.02)	0.181	-0.02 (0.02)	0.497	-0.04 (0.02)	0.125
	Reward * Session3		-0.21 (0.06)	0.001	-0.03 (0.02)	0.207	-0.02 (0.02)	0.530	-0.03 (0.02)	0.181
	Reward * Session4		-0.19 (0.06)	0.002	-0.05 (0.02)	0.063	-0.01 (0.02)	0.597	-0.03 (0.02)	0.199
	Reward * Session5		-0.18 (0.06)	0.003	-0.03 (0.02)	0.246	0.01 (0.02)	0.788	-0.04 (0.02)	0.068
	Transition * Session2		-0.08 (0.06)	0.199	-0.03 (0.02)	0.194	0 (0.02)	0.948	-0.01 (0.02)	0.834
	Transition * Session3		-0.14 (0.06)	0.026	0.02 (0.02)	0.331	0.03 (0.02)	0.265	0.01 (0.02)	0.738
	Transition * Session4		-0.17 (0.06)	0.006	0 (0.02)	0.949	-0.03 (0.02)	0.298	0.01 (0.02)	0.714
	Transition * Session5		-0.05 (0.06)	0.451	0.02 (0.02)	0.480	0.01 (0.02)	0.801	0.01 (0.02)	0.759
	Reward * Transition * Session2		0.12 (0.06)	0.067	-0.02 (0.02)	0.314	0.01 (0.02)	0.675	0.05 (0.02)	0.063
	Reward * Transition * Session3		-0.1 (0.06)	0.119	-0.01 (0.02)	0.637	0.01 (0.02)	0.626	0.02 (0.02)	0.347
	Reward * Transition * Session4		-0.05 (0.06)	0.388	0.01 (0.02)	0.668	0.01 (0.02)	0.586	0 (0.02)	0.939
	Reward * Transition * Session5		0.01 (0.06)	0.821	0.01 (0.02)	0.731	-0.01 (0.02)	0.761	0 (0.02)	0.938

### Computational model

We then fitted the seven model parameters (bMB, bMF, β_2_, α_1_, α_2_, λ, *p*) of the model variant [60] to the behavioral choice data and found the best fitting parameters to be reasonably consistent (i.e., within the 25^th^-75^th^ percentiles) with those obtained by Daw et al. [1] (**S3 Table**).

**Fig 5:**
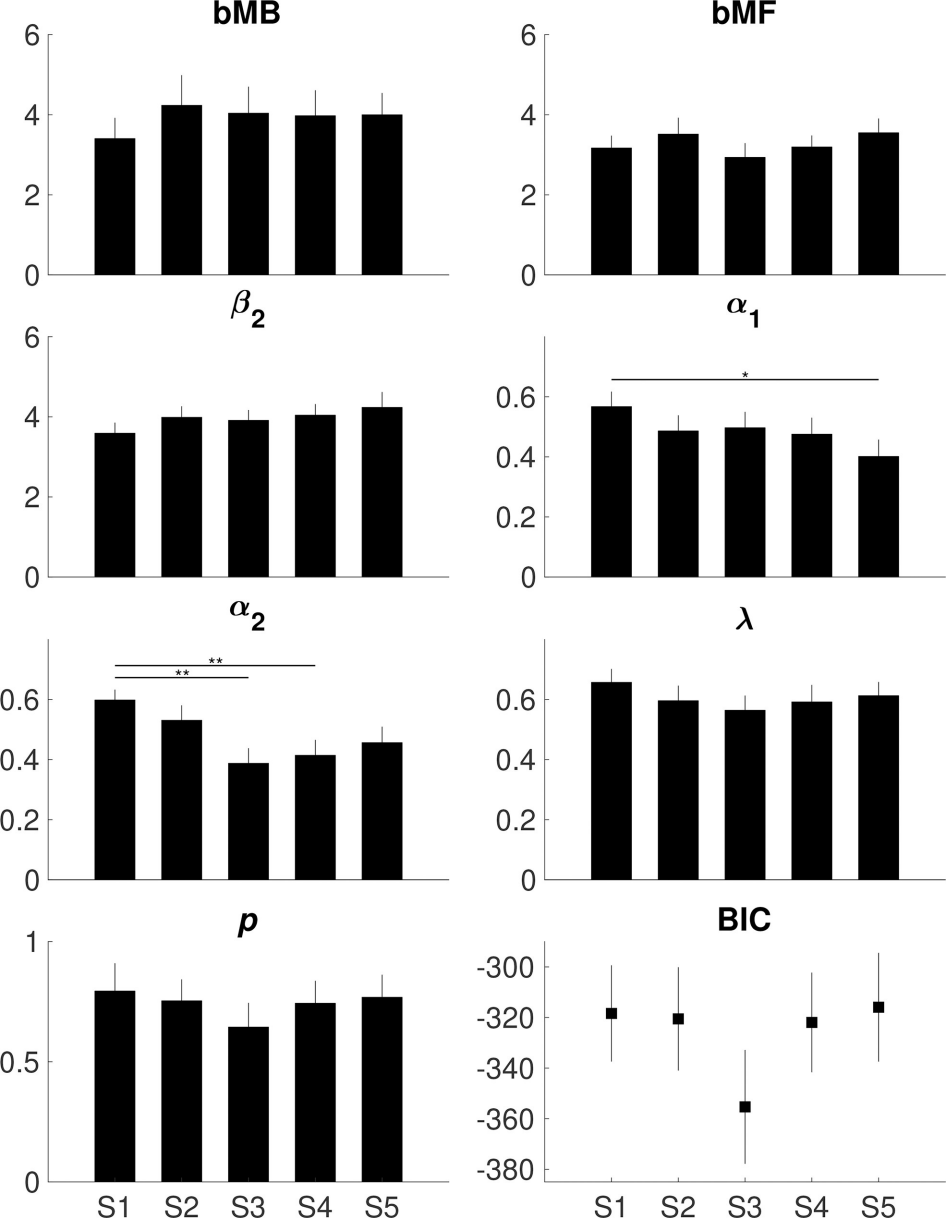
Model parameters. Estimates of the seven parameters (bMB, bMF, β_2_, α_1_, α_2_, λ, and *p*) per session (S1-S5). Error bars represent standard error of the mean. The only convincing training effects were found on α_1_ and α_2_ learning rates as assessed using repeated measures ANOVA. **BIC.** Goodness of model fit as evidenced by the subject-specific BIC was not affected by training; the smaller the BIC the better the fit. See **Table 3** for statistics.

No training effects were observed on MB control (bmB) and MF control (bMF) indicating no support for our main hypothesis that task training changes the relative strength between the two systems. MB control (bMB) was slightly stronger compared to MF control (bMF) across all sessions (t-test F1,_98_ = 2.13, p = 0.036), supporting the assumption that participants slightly more relied on MB strategies (**Table 3, Fig 5**).

**Table 3:** Model parameters. Training effects on the seven parameters (bMB, bMF, β_2_, α_1_, α_2_, λ, *p*) and BIC assessed using repeated measures ANOVA. See **Fig 5** for illustration.

		bMB	bMF	β_2_	α_1_	α_2_	Λ	P	BIC
**Main effects**	**F**_**4,76**_	0.47	1.17	0.94	2.52	5.27	1.24	0.93	1.39
	**p-value**	0.755	0.331	0.448	**0.048**	**0.001**	0.300	0.450	0.247
**Post-hoc**	**S1 vs. S2**	1.000	1.000	1.000	1.000	1.000	1.000	1.000	1.000
	**S1 vs. S3**	1.000	1.000	1.000	1.000	**0.002**	0.357	0.777	0.633
	**S1 vs. S4**	1.000	1.000	1.000	0.857	**0.009**	1.000	1.000	1.000
	**S1 vs. S5**	1.000	1.000	0.651	**0.024**	0.095	1.000	1.000	1.000
	**S2 vs. S3**	1.000	0.897	1.000	1.000	0.089	1.000	1.000	0.799
	**S2 vs. S4**	1.000	1.000	1.000	1.000	0.320	1.000	1.000	1.000
	**S2 vs. S5**	1.000	1.000	1.000	1.000	1.000	1.000	1.000	1.000
	**S3 vs. S4**	1.000	1.000	1.000	1.000	1.000	1.000	1.000	0.926
	**S3 vs. S5**	1.000	0.725	1.000	0.739	1.000	1.000	1.000	0.480
	**S4 vs. S5**	1.000	1.000	1.000	1.000	1.000	1.000	1.000	1.000

The remaining parameters also did not sufficiently argue for a shift between MB and MF control. While the parameters β_2_, λ, and *p* revealed no training effects, significant training effects were found on α_1_ and α_2_ learning rates, which decreased across sessions (repeated measures ANOVA: α_1_ F_4,76_ = 2.52, p = 0.048; α_2_ F_4,76_ = 5.27, p = 0.001). Hence, even if changes in learning rates may indicate some changes within the MF or within the MB system, they cannot be assigned to the balance or the relative expression between them. For example, increases in α_1_/ α_2_ might represent some change in the MF/MB system, and might indicate that participants consider more MF/MB strategies in the LME regression, yet it does not provide sufficient evidence to conclude that there is a change in the expression of MF relative to MB, or vice versa. Goodness of model fit was also not affected by training as evidenced by the subject-specific BIC per session (F_4,76_ = 1.39, p = 0.247) (**Table 3, Fig 5**), suggesting that there was no evidence of training-induced systematic changes in decision-making strategies not captured by the model. Across sessions, some of the parameters correlated weakly with the changes in 1^st^ and 2^nd^ stage response times as expected from the training patterns (**Table 4**). There were no significant correlations between the model parameters and NIRS responses to any of the critical trial conditions (those that were preceded by a rare/common trial, those that were rewarded/unrewarded, all p > 0.05, **S5 Table**), indicating that that NIRS responses did not inform on the behavioral changes captured by the model.

**Table 4:** Correlation between model parameters with response times and reward rates. Shown are the correlations between the seven parameters (bMB, bMF, β_2_, α_1_, α_2_, λ, *p*) with 1^st^ and 2^nd^ stage response times (RT) and reward rates as assessed using Pearson product moment correlation.

		bMB	bMF	β_2_	α_1_	α_2_	Λ	P
**1**^**st**^ **stage RT**	**r**	-0.142	-0.355	-0.266	0.313	0.086	0.046	-0.244
	**p-value**	0.159	**0.000**	**0.007**	**0.002**	0.397	0.647	**0.015**
**1**^**st**^ **stage RT**	**r**	-0.156	-0.214	-0.323	0.381	0.341	0.183	-0.177
	**p-value**	0.122	**0.033**	**0.001**	**0.000**	**0.001**	0.068	0.077
**Reward**	**r**	0.126	0.302	0.285	-0.087	0.002	0.032	0.159
	**p-value**	0.212	**0.002**	**0.004**	0.387	0.987	0.752	0.113

**Fig 6:**
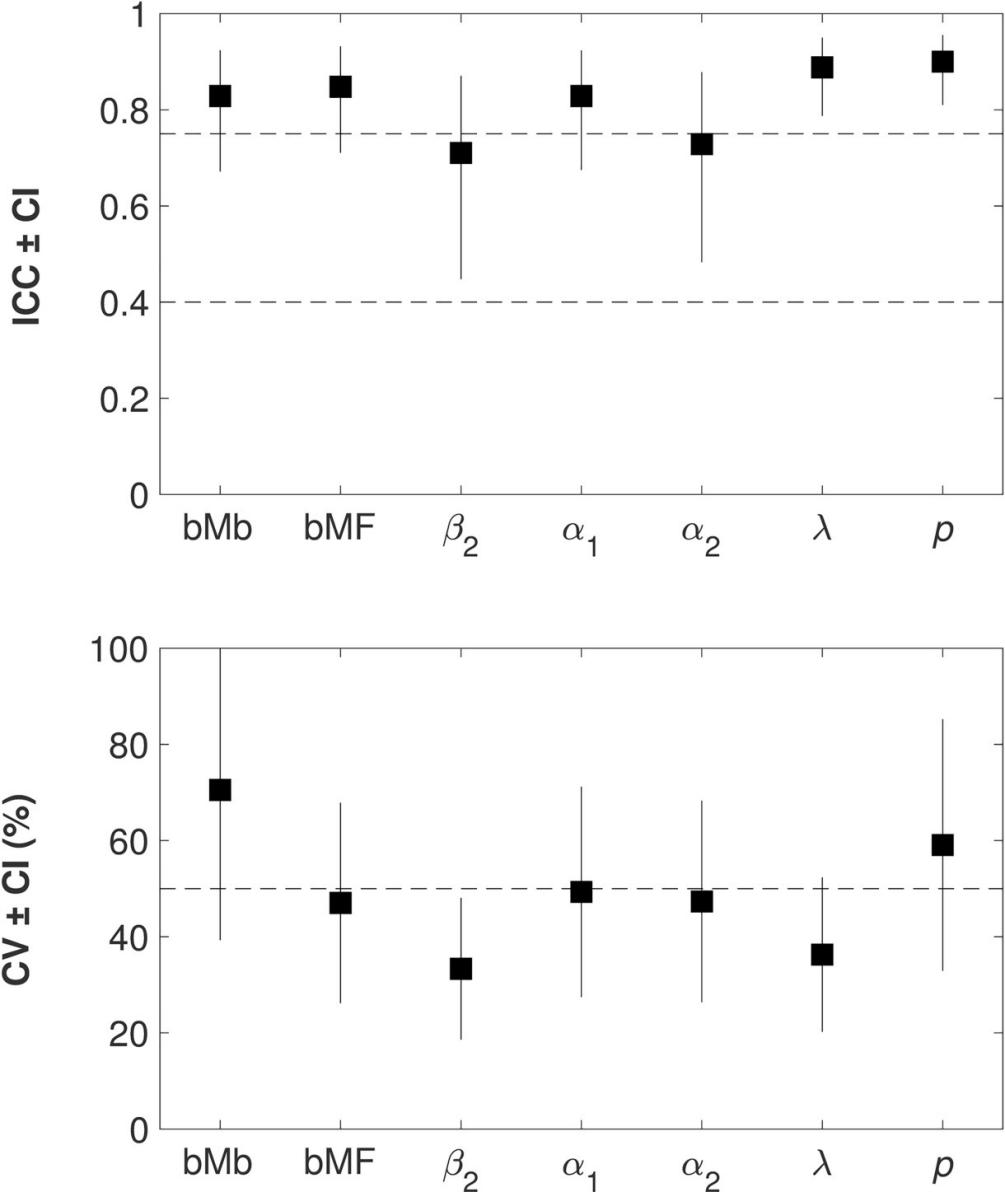
Test-retest reliability and repeatability of model parameters. Intraclass Correlation Coefficients (ICC < 0.4, 0.4–0.75, > 0.75 reflecting poor, moderate and excellent reliability [65]) and Coefficients of Variation (CV) of the seven parameters (bMB, bMF, β_2_, α_1_, α_2_, λ, *p*). See **Table 5** for statistics.

Test-retest reliability was moderate to high for all parameters, bMB (ICC = 0.83), bMB (ICC = 0.85), β_2_ (ICC = 0.71), α_1_ (ICC = 0.83), α_2_ (ICC = 0.73), λ (ICC = 0.89), *p* (ICC = 0.90), whereas test-retest repeatability was low for all parameters, bMB (CV = 71%), bMF (CV = 47%), β_2_ (CV = 33%), α_1_ (CV = 49%), α_2_ (CV = 47%), λ (CV = 36%), *p* (CV = 59%) (**Table 5, Fig 6**). The ICC results suggest that the two-step task has potential as behavioral marker for individual variation in performance, whereas the low degree of precision indicates that inter-subject variation was similar compared to intra-subject variation.

**Table 5:** Test-retest reliability and repeatability of model parameters. Intraclass Correlation Coefficients (ICC) assessing reliability and Coefficients of Variation (CV) assessing repeatability of the seven parameters (bMB, bMF, β_2_, α_1_, α_2_, λ, *p*). Upper (UB) and lower bounds (LB) of confidence intervals (CI). See **Fig 6** for illustration.

		bMB	bMF	β_2_	α_1_	α_2_	Λ	P	All
**ICC**	**UB**	0.92	0.93	0.87	0.92	0.88	0.95	0.96	0.96
	**ICC**	**0.83**	**0.85**	**0.71**	**0.83**	**0.73**	**0.89**	**0.90**	**0.95**
	**LB**	0.67	0.71	0.45	0.67	0.48	0.79	0.81	0.93
	**p-value**	**0.000**	**0.000**	**0.000**	**0.000**	**0.000**	**0.000**	**0.000**	**0.000**
**CV**	**UB**	100%	68%	48%	71%	68%	52%	85%	154%
	**CV**	**71%**	**47%**	**33%**	**49%**	**47%**	**36%**	**59%**	**106%**
	**LB**	39%	26%	19%	27%	26%	20%	33%	59%

### Simulating the relation between LME and modelling

To better understand how the LME results (suggesting training effects on MF and MB control) related to results from the computational model (suggesting no training effects on MF and MB control), we speculated that even if the computational model fully captures the learning system, choices are not only influenced by the balance between MF-MB control, but also by other model parameters. We simulated choice data with different parameter values, to understand each parameter’s independent impact on the LME. This suggested that the LME is capturing changes in all seven parameters differently (**S1 and S2 Figs, S6 Table**). Effects of ‘reward’ were primarily positively correlated with changes in the parameters bMF (correlation index MF_CI_ = 0.992), α_1_ (MF_CI_ = 1.000), λ (MF_CI_ = 0.970) and p (MFCCI = -0.742), i.e., a decrease in any of these parameter values results in decreasing LME coefficients for MF control; while ‘reward * transition’ interactions seemed to be primarily positively correlated with changes in the parameters bMB (MB_CI_ = 0.983), β_2_ (MB_CI_ = 0.995) and α_2_ (MB_CI_ = 0.996), i.e., a decrease in any of these parameter values results in decreasing LME coefficients for MB. Note that magnitudes of these indices should only be interpreted in the context of the simulation. In summary, these findings indicate that even under the assumption that the model fully captures the cognitive system mediating learning in this task, then LME one-step effects not only reflect contribution of the MF and MB systems, but also parametric changes within the two systems. This means that interpreting the ‘reward’ and ‘reward * transition’ coefficients as directly indexing MF and MB control may be misleading. One interpretation of our discrepant results therefore is that the LME results capture changes in α_1_ and α_2_, which did change between sessions. Because these two parameters have no direct relation to either MF or MB control or the balance between the two systems, this not support an assumption of training effects on MF or MB strategies. To corroborate these conclusions, we provide an illustration that the regression coefficients based on our simulations allow reconstructing the actual LME pattern that we observe from our fitted computational model coefficients (**S3 Fig**). It should however be noted that the method presented here designed to assess how LME regression captures the seven model parameters, cannot be reversed, i.e., it the model parameters itself cannot be recovered. The method can therefore only be applied and interpreted in the context of the LME.

### Power analysis

Since the presented results are negative findings, we performed a post-hoc power analysis using a previously published distribution of the parameters bMF and bMB [68]. Under the assumption that our training changes parameters linearly over the five sessions, that it does not change the variance in the parameters over individuals, and that the test-retest-reliability of the parameters is zero (i.e., that between-subject variation in the parameters is not due to stable traits), then our sample size of N = 20 would have been sufficient to detect an at least 80% change in bMF and an at least 120% change in bMB with 80% power at an alpha level of 5%. Assuming a test-retest reliability of 0.5, we had sufficient power to detect a 60% change in bMF and an 85% change in bMB; and at a test-retest reliability of 0.8, these values were 35% change in bMF and a 55% change in bMB.

## Discussion

In this paper, we tested a hypothesis that training humans on a two-step task reduces the influence of MF control whilst strengthening the influence of MB control. Such training may be relevant for assessing psychiatric conditions including compulsion or addiction, because of their reported association with an overreliance on habits [24]. Our results show that the two-step task reliably assesses individual MF and MB behavior but that training on the two-step task in its current form does not support a shift in the balance between the two systems. Training on the two-step task may thus require further adaptations in order to reduce MF control or compensate for deficits in goal-directed choice. Although the current study was conducted in healthy subjects and may therefore not be directly generalizable to psychiatric populations with premorbid, i.e., pre-training, deficits in MB control, our results may contribute to the current debate how the two-step could be adjusted to be used as training tool and to advance its application in the trans-diagnostic evaluation of psychiatric conditions [43,67].

### Reliability of MF or MB control

Results of the behavioral model indicated higher test-retest reliability for the two-step task (overall ICC = 0.95, **Table 5, Fig 6**) than previously reported in a literature review (approx. mean ICC = 0.7) [69]. Although the purpose of the present study was the evaluation of training that was supposed to change behavior and thus requires caution in the interpretation of reliability, our findings suggest that the two-step task has potential as a behavioral marker to characterize individual behavior. The high reliability was associated with low precision (overall CV = 106%, **Table 5, Fig 6**) indicating that the standard deviation exceeded the mean value, in other words, that inter-subject variation was similar compared to intra-subject variation. Together, this suggests that the model does reflect individual variation but is not precise.

### No substantial change in MF or MB control via training

Results of the behavioral model suggest that training on the two-step task in its current form does not affect the balance between MF and MB control, as exemplified by a relatively stable pattern of the bMF and bMB parameters across sessions (**Table 3, Fig 5**). The only convincing training effects were reflected in decreasing α_1_ and α_2_ learning rates. This indicates that the degree to which participants incorporated new information decreased as task training progressed. Considering these modeling results and our simulations on the relation of model parameters and choice behaviors, the LME effects on behavioral data and the brain data (**Table 2, Fig 4**) most likely do not reflect changes in the balance between MF and MB control, nor in the individual systems, but merely capture changes in α_1_ and α_2_ based on the parametric mapping on LME (**S1 Fig, S4 Table**). Together, these findings suggest that training on the two-step task induced no substantial changes in decision strategies besides affecting learning rates. Although the results support some correspondence between behavioral choice and ilPFC, our results do not support a previous hypothesis that ilPFC arbitrates between the MF and MB system [27]. As a limitation, our power analysis indicates that a larger sample would be required to find small training effects (e.g. parameter change smaller than 50% at a parameter test-retest reliability of r = 0.8).

### Comparison with previous training study

A previous training study utilizing the same two-step task by Economides et al. [9] reported evidence of training effects. Training increased MB control (as evidenced by an increased α learning rate, an increased weighting parameter ω and increased ‘reward * transition’ interactions), while leaving MF control unaffected (as evidenced by unchanged ‘reward’ effects). These behavioral changes were however observed following the concurrent introduction of a secondary load task, and the authors conjectured that the addition of load may have been necessary to expose training-induced changes in behavior in the two-step task. There are also several other possible explanations for this disparity. One likely candidate is the difference in training intervals. Economides et al. [9] trained subjects over three consecutive days, whereas the present study trained subjects over five days separated by a week. Another reason might be the difference in training intensity. Economides et al. [9] trained subjects on 768 trials, whereas we trained subjects on 1005 trials, almost one-third more trials. A third reason might be differences in statistical analysis methodology. Economides et al. [9] did not test for interactions between reward, transition and session in the LME and made use of an additional slope parameter sigma (σ) that allowed the weighting parameter ω to shift across training sessions when fitting data across all sessions; notably an implementation of the sigma (σ) parameter in our model did not change overall results (analysis not included in this article).

### Interpreting the lack of changes in MF and MB control

Within each session of the present study, participants followed slightly more MB strategies, as indicated by a median ratio between bMB and bMF of 1.07 (p = 0.041) (compared to a median weighting parameter ω of 0.39 indicating more reliance on MF strategy reported by Daw et al. [1], **S3 Table**). Hence, participants were able to establish an internal model of the task by considering the dynamic interactions between rewards and transitions, although training did not strengthen that internal model.

The missing training effect might be due to a natural re-equilibration of the balance to its default setting, i.e., the MF system, which is less computationally demanding. The arbitrator responsible for inhibiting the default habitual control and deliberating the MB system [27] may have become weaker towards the end of training due to habituation. Additional cofounders like tiredness and monotony induced by the high number of repetitions may have favored less effortful MF strategies, as supported by the progressively faster response times observed across sessions (**Table 1, Fig 3**). Demotivation or devaluation may also be justified by the missing trade-off between performance accuracy and reward rates (**Table 1, Fig 3**). It is well-established that payoffs in the two-step task do not differ between performance of strictly MF versus strictly MB agents or even agents who chose randomly [43,67]. These findings suggest that the stochasticity of the two-step task imposes a low ceiling on achievable performance, preventing MB control from outperforming simple MF strategies [43]. It might have therefore been rational for participants to not invest in the higher cognitive costs of MB strategies, as they did not pay off.

The missing training effect might also point to the employment of a third decision-making strategy, namely sophisticated automatization, that is distinct from pure MF and MB learning [43]. Previous simulations suggested that the two-step paradigm may or even should promote such a third control system [43]. Faced with recurrent transitions there might be an increased incentive to deconstruct the task and identify stimuli for automatized responses. This may produce a behavior that mimics goal-directed planning but in fact arises as a fixed mapping of limited states matched with habitual response and automatable strategies [13,43]. This kind of automatization could indeed be beneficial as it may render MB control less susceptible to distraction [9]. Arguments for automatization may thus that it reduces the computational cost associated with MB planning, making MB reasoning more efficient, although not explicitly impacting the balance between MF and MB decision processes.

To enable training effects on MB learning while also allowing for some degree of automatization, several task adaptations have been proposed, such as increasing payoff attractiveness by enhancing the trade-off been performance accuracy and reward, sharpening contrasts between transition and reward probabilities, increasing complexity of decision trees while compensating with simpler transitions, masking high frequent repetitions by alternated task settings to reduce the burden of automatization [43,67]. Using such incentives to boost model-based control has also been suggested to be a useful intervention in a range of personality traits and latent psychiatric symptom constructs [70].

### Conclusion

Previous evidence suggests that an imbalance between MF and MB control may be a common mechanism in various psychiatric disorders. The potential to rebalance such decision strategies through task training therefore remains a promising therapeutic approach. The present study suggests that training on the two-step task in its current form does not change the balance between MF and MB control. An evaluation in psychiatric populations is required to assess whether the present results can be translated into a trans-diagnostic framework [50].

## Supporting information

S1 Table fNIRS setup. Sources, detectors, and channels for selected regions of interest (ROIs) representing the model-free system (vmPFC, dlPFC) and the arbitrator (ilPFC) illustrated in **Fig 2** according to the International 10–20 system [71] and the MNI (Montreal Neurological Institute) coordinates [72]. Sources (S) Detectors (D).
(DOCX)

S2 Table Bayesian model comparison. Results of a Bayesian comparison of eight model variants of the original hybrid model by Daw et al. [1] as implemented in the Emfit toolbox [73] that account for differences in model complexity. Each model was assessed across all five training sessions. Model variants may consist of separate parameters for 1^st^ and 2^nd^ stage choices (α_1/2_ = learning rate; β_1/2_ = softmax inverse temperature), an eligibility trace (λ), first-order perseveration (*p*), two separate betas, one for the model-free system (bMF) and for the model-based system (bMB),or a weighting parameter (ω) that determines the balance between model-free (ω = 0) and model-based (ω = 1) control. In simpler models, parameters were fixed between 1^st^ and 2^nd^ stage choices. Model llm2b2alr is the original hybrid model by Daw et al. [1]. Bold-face denotes the winning model variant ll2bmfbmb2alr based on the lowest integrated Bayesian information criterion (iBIC) score that was used in the present analysis.
(DOCX)

S3 Table Best-fitting parameter estimates. Best-fitting parameter estimates (β_1_, β_2_, α_1_, α_2_, λ, ω and *p*) shown as median plus 25^th^ and 75^th^ percentile across sessions S1-S5 obtained with the model variant in the present analysis in comparison with the estimates obtained with the original model by Daw et al. [1]. Note that the parameter *p* has a different scale in the model variant.
(DOCX)

S4 Table Distribution of Simulated parameter values. Simulation data were generated for each of the seven parameters (untransformed values) within the distribution of the untransformed values obtained from the actual data (5^th^, 25^th^, 50^th^, 75^th^, 95^th^ percentile, across sessions S1-S5) while keeping the remaining parameters constant at the median.
(DOCX)

S5 Table Correlation between model parameters and NIRS responses. Listed are the Pearson correlations between the model parameters (bMB, bMF, β_2_, α_1_, α_2_, λ, p) with the averaged NIRS responses within critical trials (those that were preceded by a rare/common trial, those that were rewarded/unrewarded) on the single subject level across all sessions. The results indicated no significant correlations.
(DOCX)

S6 Table Simulated correlation indices. Listed are the inferred MF and MB correlation indices (MF_CI_ and MB_CI_) for each parameter (bMB, bMF, β_2_, α_1_, α_2_, λ, p) approximating the parameter-specific change in LME coefficients for MF control (‘reward’ effect) and MB control (‘reward * transition’ interaction). Positive versus negative correlation indices indicate that parameters are positively versus negatively correlated with LME coefficients. Note that the magnitudes of these indices should only be interpreted in the context of the simulation.
(DOCX)

S1 Fig LME main effects per session. Each bar represents the stay probability (p(stay)) or mean tHb response across all participants for each session. For each session, bars from left to right represent R^+^C, R^+^U, R^-^C, R^-^U (R+ = rewarded vs. R- = unrewarded, C = common vs. U = uncommon, as detailed in **Fig 4**) Error bars represent standard error of the mean. See **Table 2** for statistics.
(TIF)

S2 Fig Effects of independent parameter changes on LME. Results of the simulation assessing independent changes of the parameters (bMB, bMF, β_2_, α_1_, α_2_, λ, p) on LME. **(Top)** Inferred LME regression main effects. For each percentage change, bars from left to right represent R^+^C, R^+^U, R^-^C, R^-^U (R+ = rewarded vs. R- = unrewarded, C = common vs. U = uncommon, as detailed in **Fig 4**) **(Bottom)** Inferred LME coefficients representing parameter-specific changes in LME coefficients for MF control (‘reward’ effect) and MB control (‘reward * transition’ interaction).
(TIF)

S3 Fig Correlation indices used to reconstruct LME coefficients. Illustration of a simple approximation to reconstruct the patterns of the MF (‘reward’ effect) and MB (‘reward * transition’ interaction) coefficients for comparison with the actual LME. Reconstruction was done by multiplying the correlation indices (MF_CI_ and MB_CI_, **S6 Table**) with the actual parameter values (bMB, bMF, β_2_, α_1_, α_2_, λ, p). **(Left)** To reconstruct the MF coefficients, the mean values of the parameters primarily affecting MF control (bMF, α_1_, and λ) multiplied with the corresponding MF_CI_ per session were summed for illustration. **(Right)** To reconstruct the MB coefficients, the mean values of the parameters primarily affecting MB control (bMB, β_2_, and α_2_,) multiplied with the corresponding MB_CI_ per session were summed for illustration. According to the actual LME results, data are shown in comparison with the reference session S1.
(TIF)
