# Supplementary material for: No substantial change in the balance between model-free and model-based control via training on the two-step task
Source: PLoS Comput Biol. 2019 Nov 14;15(11):e1007443. doi: 10.1371/journal.pcbi.1007443 (PMC6855413; doi:10.1371/journal.pcbi.1007443)
Supplement: S1 Table — Sources, detectors, and channels for selected regions of interest (ROIs) representing the model-free system (vmPFC, dlPFC) and the arbitrator (ilPFC) illustrated in Fig 2 according to the International 10–20 system [71] and the MNI (Montreal Neurological Institute) coordinates [72]. Sources (S) Detectors (D). (DOCX) [file pcbi.1007443.s001.docx]

|  | **10-20 system** | | **MNI** | | |
| --- | --- | --- | --- | --- | --- |
|  |  |  | **x** | **Y** | **z** |
| **vmPFC** | S1 | Fpz | 3.0 | 67.6 | -3.0 |
|  | D1 | Fp1 | -22.1 | 67.6 | -6.5 |
|  | S2 | Fp2 | 25.9 | 68.1 | -7.1 |
|  | D2 | AFz | 2.9 | 64.5 | 25.9 |
| **dlPFC** | S5 | FC5 | -61.1 | 12.9 | 20.5 |
|  | S7 | FC6 | 62.9 | 11.2 | 21.1 |
|  | D7 | FFC5h | -51.2 | 29.5 | 28.2 |
|  | D8 | FFC6h | 54.5 | 29.4 | 29.6 |
|  | S8 | FC4 | 50.1 | 16.5 | 46.5 |
|  | S6 | FC3 | -48.2 | 16.3 | 45.6 |
| **ilPFC** | D3 | F7 | -52.0 | 38.0 | -9.8 |
|  | D5 | F8 | 54.0 | 37.8 | -9.6 |
|  | D4 | FFC7h | -56.8 | 26.2 | 3.6 |
|  | D6 | FFC8h | 58.8 | 25.4 | 5.1 |
|  | S3 | F5 | -51.7 | 40.4 | 10.6 |
|  | S4 | F6 | 53.8 | 39.6 | 11.7 |
