# Supplementary material for: No substantial change in the balance between model-free and model-based control via training on the two-step task
Source: PLoS Comput Biol. 2019 Nov 14;15(11):e1007443. doi: 10.1371/journal.pcbi.1007443 (PMC6855413; doi:10.1371/journal.pcbi.1007443)
Supplement: S2 Table — Results of a Bayesian comparison of eight model variants of the original hybrid model by Daw et al. [1] as implemented in the Emfit toolbox [73] that account for differences in model complexity. Each model was assessed across all five training sessions. Model variants may consist of separate parameters for 1st and 2nd stage choices (α1/2 = learning rate; β1/2 = softmax inverse temperature), an eligibility trace (λ), first-order perseveration (p), two separate betas, one for the model-free system (bMF) and for the model-based system (bMB),or a weighting parameter (ω) that determines the balance between model-free (ω = 0) and model-based (ω = 1) control. In simpler models, parameters were fixed between 1st and 2nd stage choices. Model llm2b2alr is the original hybrid model by Daw et al. [1]. Bold-face denotes the winning model variant ll2bmfbmb2alr based on the lowest integrated Bayesian information criterion (iBIC) score that was used in the present analysis. (DOCX) [file pcbi.1007443.s002.docx]

| **Models** | **Parameters** | **iBIC (x 10^4^)** | **No. Parameters** |
| --- | --- | --- | --- |
| llbmfbmb2alcr | bmb bmf β^2^ α^1^ α^2^ λ p | 3.9540 | 7 |
| llbmbar | bmb bmf α p | 3.9219 | 4 |
| llbmfalr | bmf α λ p | 3.8814 | 4 |
| ll2bmfalr | bmf β^2^ α λ p | 3.8693 | 5 |
| ll2bmf2alr | bmf β^2^ α^1^ α^2^ λ p | 3.8629 | 6 |
| llbmfbmbalr | bmb bmf α λ p | 3.8202 | 5 |
| ll2bmfbmbalr | bmb bmf β^2^ α λ p | 3.8034 | 6 |
| llm2b2alr | β^1^ β^2^ α^1^ α^2^ λ ω p | 3.7970 | 7 |
| ll2bmfbmb2alr | bmb bmf β^2^ α^1^ α^2^ λ p | **3.7948** | 7 |
