# Supplementary material for: No substantial change in the balance between model-free and model-based control via training on the two-step task
Source: PLoS Comput Biol. 2019 Nov 14;15(11):e1007443. doi: 10.1371/journal.pcbi.1007443 (PMC6855413; doi:10.1371/journal.pcbi.1007443)
Supplement: S3 Table — Best-fitting parameter estimates (β1, β2, α1, α2, λ, ω and p) shown as median plus 25th and 75th percentile across sessions S1-S5 obtained with the model variant in the present analysis in comparison with the estimates obtained with the original model by Daw et al. [1]. Note that the parameter p has a different scale in the model variant. (DOCX) [file pcbi.1007443.s003.docx]

|  | | **bMB** | **bMF** | 𝜷_2_ | 𝜶_1_ | 𝜶_2_ | λ | *p* | 𝜔 |
| --- | --- | --- | --- | --- | --- | --- | --- | --- | --- |
| **Model variant** | 25^th^ | 2.09 | 2.65 | 3.34 | 0.33 | 0.40 | 0.43 | 0.43 |  |
|  | **Median** | **3.31** | **3.09** | **3.92** | **0.48** | **0.46** | **0.61** | **0.72** |  |
|  | 75^th^ | 5.71 | 3.69 | 4.62 | 0.65 | 0.53 | 0.70 | 1.05 |  |
| **Original model** | 25^th^ |  |  | 2.69 | 0.46 | 0.21 | 0.41 | 0.02 | 0.29 |
|  | **Median** |  |  | **3.69** | **0.54** | **0.42** | **0.57** | **0.11** | **0.39** |
|  | 75^th^ |  |  | 5.16 | 0.87 | 0.71 | 0.94 | 0.22 | 0.59 |
