# Supplementary material for: No substantial change in the balance between model-free and model-based control via training on the two-step task
Source: PLoS Comput Biol. 2019 Nov 14;15(11):e1007443. doi: 10.1371/journal.pcbi.1007443 (PMC6855413; doi:10.1371/journal.pcbi.1007443)
Supplement: S4 Table — Simulation data were generated for each of the seven parameters (untransformed values) within the distribution of the untransformed values obtained from the actual data (5th, 25th, 50th, 75th, 95th percentile, across sessions S1-S5) while keeping the remaining parameters constant at the median. (DOCX) [file pcbi.1007443.s004.docx]

|  | **bMB** | **bMF** | **𝜷_2_** | **𝜶_1_** | **𝜶_2_** | **λ** | ***p*** |
| --- | --- | --- | --- | --- | --- | --- | --- |
| **5^th^** | 0.41 | 0.32 | 0.86 | -1.63 | -1.99 | -0.57 | 0.15 |
| **25^th^** | 0.69 | 0.83 | 1.16 | -0.84 | -0.44 | -0.28 | 0.43 |
| **Median** | **1.06** | **1.09** | **1.31** | **-0.10** | **-0.23** | **0.49** | **0.72** |
| **75^th^** | 1.65 | 1.26 | 1.49 | 0.66 | 0.13 | 0.96 | 1.05 |
| **95^th^** | 2.04 | 1.78 | 1.73 | 1.51 | 1.42 | 2.60 | 1.32 |
