# Supplementary material for: No substantial change in the balance between model-free and model-based control via training on the two-step task
Source: PLoS Comput Biol. 2019 Nov 14;15(11):e1007443. doi: 10.1371/journal.pcbi.1007443 (PMC6855413; doi:10.1371/journal.pcbi.1007443)
Supplement: S5 Table — Listed are the Pearson correlations between the model parameters (bMB, bMF, β2, α1, α2, λ, p) with the averaged NIRS responses within critical trials (those that were preceded by a rare/common trial, those that were rewarded/unrewarded) on the single subject level across all sessions. The results indicated no significant correlations. (DOCX) [file pcbi.1007443.s005.docx]

| **Correlation between model parameters and average NIRS response to RARE trials** | | | | | | |
| --- | --- | --- | --- | --- | --- | --- |
|  | ilPFC | | vmPFC | | dlPFC | |
|  | r | p-value | r | p-value | r | p-value |
| bMB | -0.154 | 0.126 | 0.040 | 0.692 | -0.149 | 0.138 |
| bMF | -0.026 | 0.798 | 0.055 | 0.586 | -0.023 | 0.817 |
| β2 | -0.157 | 0.118 | 0.087 | 0.391 | -0.136 | 0.179 |
| ɑ1 | 0.021 | 0.839 | -0.247 | 0.013 | 0.010 | 0.924 |
| ɑ2 | 0.103 | 0.306 | -0.143 | 0.155 | 0.064 | 0.524 |
| ƛ | 0.021 | 0.838 | -0.047 | 0.643 | 0.021 | 0.839 |
| p | -0.034 | 0.737 | -0.004 | 0.971 | -0.021 | 0.835 |
|  |  |  |  |  |  |  |
| **Correlation between model parameters and average NIRS response to COMMON trials** | | | | | | |
|  | ilPFC | | vmPFC | | dlPFC | |
|  | r | p-value | r | p-value | r | p-value |
| bMB | -0.033 | 0.747 | 0.023 | 0.820 | -0.040 | 0.694 |
| bMF | -0.011 | 0.913 | -0.013 | 0.894 | -0.031 | 0.758 |
| β2 | -0.025 | 0.802 | 0.102 | 0.311 | -0.030 | 0.764 |
| ɑ1 | 0.118 | 0.242 | -0.128 | 0.203 | 0.111 | 0.273 |
| ɑ2 | 0.078 | 0.442 | -0.254 | 0.011 | 0.038 | 0.710 |
| ƛ | 0.040 | 0.691 | -0.093 | 0.355 | 0.029 | 0.777 |
| p | -0.026 | 0.795 | -0.027 | 0.790 | -0.042 | 0.676 |
|  |  |  |  |  |  |  |
| **Correlation between model parameters and average NIRS response to REWARDED trials** | | | | | | |
|  | ilPFC | | vmPFC | | dlPFC | |
|  | r | p-value | r | p-value | r | p-value |
| bMB | -0.144 | 0.154 | 0.000 | 0.999 | -0.152 | 0.132 |
| bMF | -0.052 | 0.609 | 0.015 | 0.879 | -0.051 | 0.615 |
| β2 | -0.159 | 0.114 | 0.078 | 0.440 | -0.136 | 0.177 |
| ɑ1 | 0.213 | 0.033 | -0.168 | 0.095 | 0.174 | 0.083 |
| ɑ2 | 0.157 | 0.120 | -0.184 | 0.066 | 0.090 | 0.375 |
| ƛ | 0.078 | 0.442 | -0.051 | 0.613 | 0.069 | 0.494 |
| p | -0.046 | 0.649 | -0.009 | 0.931 | -0.058 | 0.564 |
|  |  |  |  |  |  |  |
| **Correlation between model parameters and average NIRS response to UNREWARDED trials** | | | | | | |
|  | ilPFC | | vmPFC | | dlPFC | |
|  | r | p-value | r | p-value | r | p-value |
| bMB | 0.002 | 0.981 | 0.067 | 0.509 | -0.002 | 0.986 |
| bMF | -0.001 | 0.989 | -0.002 | 0.980 | -0.010 | 0.919 |
| β2 | 0.011 | 0.915 | 0.117 | 0.247 | 0.000 | 1.000 |
| ɑ1 | -0.096 | 0.343 | -0.156 | 0.122 | -0.071 | 0.483 |
| ɑ2 | 0.002 | 0.985 | -0.249 | 0.012 | 0.004 | 0.965 |
| ƛ | -0.042 | 0.679 | -0.111 | 0.274 | -0.040 | 0.690 |
| p | -0.007 | 0.947 | -0.018 | 0.859 | -0.005 | 0.962 |
