# Supplementary material for: No substantial change in the balance between model-free and model-based control via training on the two-step task
Source: PLoS Comput Biol. 2019 Nov 14;15(11):e1007443. doi: 10.1371/journal.pcbi.1007443 (PMC6855413; doi:10.1371/journal.pcbi.1007443)
Supplement: S6 Table — Listed are the inferred MF and MB correlation indices (MFCI and MBCI) for each parameter (bMB, bMF, β2, α1, α2, λ, p) approximating the parameter-specific change in LME coefficients for MF control (‘reward’ effect) and MB control (‘reward * transition’ interaction). Positive versus negative correlation indices indicate that parameters are positively versus negatively correlated with LME coefficients. Note that the magnitudes of these indices should only be interpreted in the context of the simulation. (DOCX) [file pcbi.1007443.s006.docx]

|  |  |  |  | **MF_CI_** | |  | **MB_CI_** | |
| --- | --- | --- | --- | --- | --- | --- | --- | --- |
| **Parameters** | **Percentiles** | **Values** | **Inferred MF coefficients** | **Pearson r** | **p-**  **value** | **Inferred MB coefficients** | **Pearson r** | **p-value** |
| **bMB** | 5th | 0.41 | 0.4575 | -0.875 | 0.052 | 0.1532 | **0.983** | **0.003** |
|  | 25th | 0.69 | 0.4635 |  |  | 0.1718 |  |  |
|  | 50th | **1.06** | 0.4569 |  |  | 0.2516 |  |  |
|  | 75th | 1.65 | 0.4517 |  |  | 0.4071 |  |  |
|  | 95th | 2.04 | 0.4396 |  |  | 0.5607 |  |  |
| **bMF** | 5th | 0.32 | 0.2314 | **0.992** | **0.001** | 0.2632 | -0.935 | 0.020 |
|  | 25th | 0.83 | 0.3630 |  |  | 0.2579 |  |  |
|  | 50th | **1.09** | 0.4600 |  |  | 0.2574 |  |  |
|  | 75th | 1.26 | 0.5319 |  |  | 0.2536 |  |  |
|  | 95th | 1.78 | 0.7507 |  |  | 0.2388 |  |  |
| **β_2_** | 5th | 0.86 | 0.4569 | -0.725 | 0.166 | 0.2323 | **0.995** | **0.000** |
|  | 25th | 1.16 | 0.4558 |  |  | 0.2531 |  |  |
|  | 50th | **1.31** | 0.4479 |  |  | 0.2579 |  |  |
|  | 75th | 1.49 | 0.4545 |  |  | 0.2670 |  |  |
|  | 95th | 1.73 | 0.4470 |  |  | 0.2801 |  |  |
| **α_1_** | 5th | -1.63 | 0.2120 | **1.000** | **0.000** | 0.2661 | -0.889 | 0.044 |
|  | 25th | -0.84 | 0.3246 |  |  | 0.2642 |  |  |
|  | 50th | **-0.1** | 0.4554 |  |  | 0.2604 |  |  |
|  | 75th | 0.66 | 0.5709 |  |  | 0.2427 |  |  |
|  | 95th | 1.51 | 0.6984 |  |  | 0.2469 |  |  |
| **α_2_** | 5th | -1.99 | 0.4547 | -0.369 | 0.541 | 0.0858 | **0.996** | **0.000** |
|  | 25th | -0.44 | 0.4593 |  |  | 0.2253 |  |  |
|  | 50th | **-0.23** | 0.4664 |  |  | 0.2530 |  |  |
|  | 75th | 0.13 | 0.4554 |  |  | 0.2976 |  |  |
|  | 95th | 1.42 | 0.4477 |  |  | 0.4485 |  |  |
| **Λ** | 5th | -0.57 | 0.3217 | **0.970** | **0.006** | 0.2580 | 0.660 | 0.225 |
|  | 25th | -0.28 | 0.3713 |  |  | 0.2604 |  |  |
|  | 50th | **0.49** | 0.4496 |  |  | 0.2560 |  |  |
|  | 75th | 0.96 | 0.5041 |  |  | 0.2615 |  |  |
|  | 95th | 2.6 | 0.5815 |  |  | 0.2631 |  |  |
| ***p*** | 5th | 0.15 | 0.4618 | -0.742 | 0.151 | 0.2655 | -0.695 | 0.193 |
|  | 25th | 0.43 | 0.4555 |  |  | 0.2545 |  |  |
|  | 50th | **0.72** | 0.4493 |  |  | 0.2560 |  |  |
|  | 75th | 1.05 | 0.4515 |  |  | 0.2583 |  |  |
|  | 95th | 1.32 | 0.4523 |  |  | 0.2521 |  |  |
