# Supplementary figures and images for: No substantial change in the balance between model-free and model-based control via training on the two-step task
Source: PLoS Comput Biol. 2019 Nov 14;15(11):e1007443. doi: 10.1371/journal.pcbi.1007443 (PMC6855413; doi:10.1371/journal.pcbi.1007443)

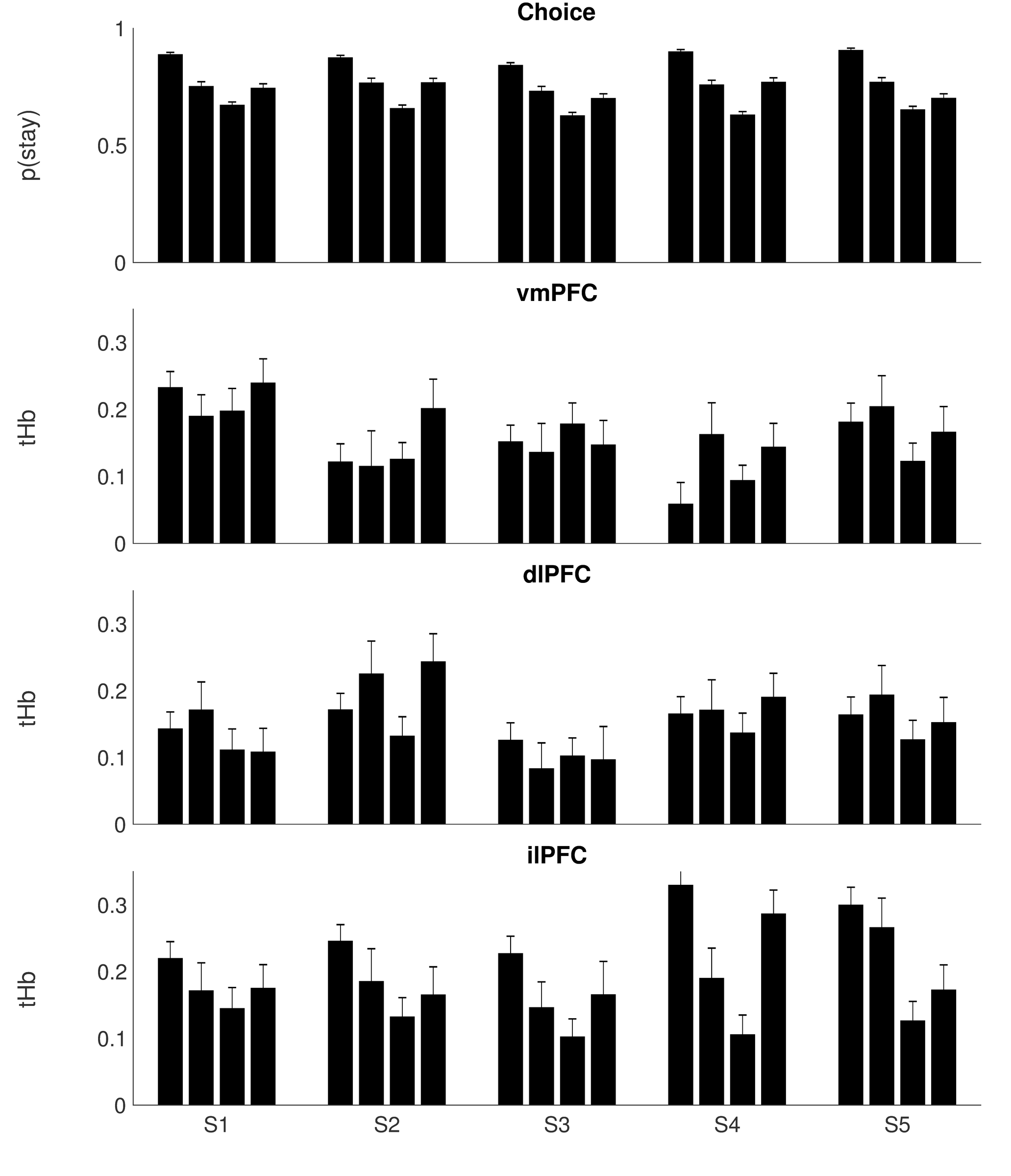

Supplement: S1 Fig — Each bar represents the stay probability (p(stay)) or mean tHb response across all participants for each session. For each session, bars from left to right represent R+C, R+U, R-C, R-U (R+ = rewarded vs. R- = unrewarded, C = common vs. U = uncommon, as detailed in Fig 4) Error bars represent standard error of the mean. See Table 2 for statistics. (TIF) [file pcbi.1007443.s007.tif]

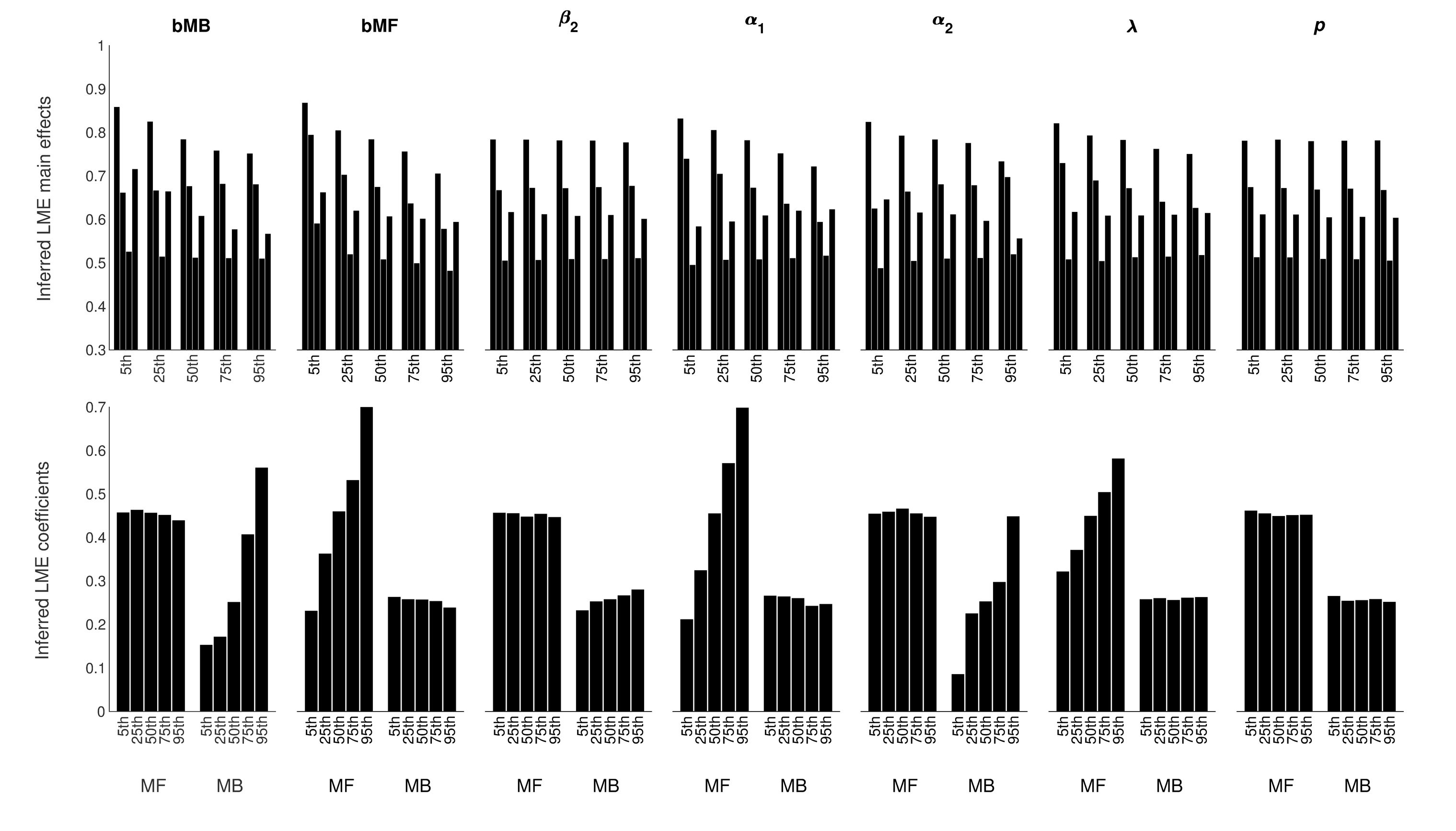

Supplement: S2 Fig — Results of the simulation assessing independent changes of the parameters (bMB, bMF, β2, α1, α2, λ, p) on LME. (Top) Inferred LME regression main effects. For each percentage change, bars from left to right represent R+C, R+U, R-C, R-U (R+ = rewarded vs. R- = unrewarded, C = common vs. U = uncommon, as detailed in Fig 4) (Bottom) Inferred LME coefficients representing parameter-specific changes in LME coefficients for MF control (‘reward’ effect) and MB control (‘reward * transition’ interaction). (TIF) [file pcbi.1007443.s008.tif]

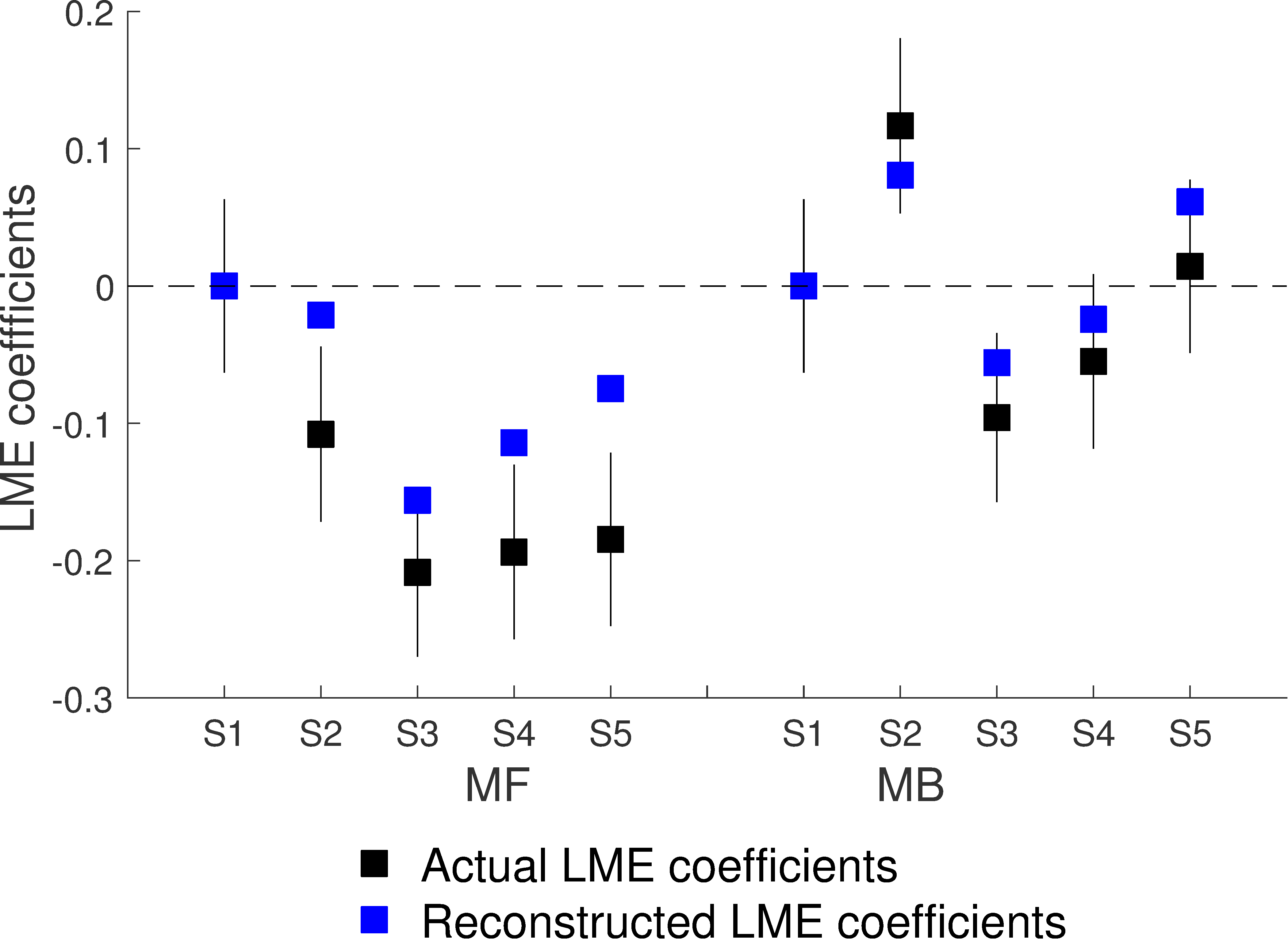

Supplement: S3 Fig — Illustration of a simple approximation to reconstruct the patterns of the MF (‘reward’ effect) and MB (‘reward * transition’ interaction) coefficients for comparison with the actual LME. Reconstruction was done by multiplying the correlation indices (MFCI and MBCI, S6 Table) with the actual parameter values (bMB, bMF, β2, α1, α2, λ, p). (Left) To reconstruct the MF coefficients, the mean values of the parameters primarily affecting MF control (bMF, α1, and λ) multiplied with the corresponding MFCI per session were summed for illustration. (Right) To reconstruct the MB coefficients, the mean values of the parameters primarily affecting MB control (bMB, β2, and α2,) multiplied with the corresponding MBCI per session were summed for illustration. According to the actual LME results, data are shown in comparison with the reference session S1. (TIF) [file pcbi.1007443.s009.tif]
